# Prevalence of HTLV-1 infection in northeast of Iran

**DOI:** 10.1186/1742-4690-12-S1-O7

**Published:** 2015-08-28

**Authors:** Reza Farid, Farhad Farid, Safai A Rezaee

**Affiliations:** 1 Department of Neurology, Mashhad University of Medical Sciences, Iran

## 

Northeast of Iran is a new endemic. Area for HTLV-1 and for the first time. Farid and his Immunology Dep at Mashhad University published paper and then Farid and safae in Aids research and human Retro virology volt 12 November 12 1996. Published prevalence of HTLV-1 in Iran several. Publication describes the prevalence of HTLV-1 in Jewish individuals born in city of Mashhad Iran. Then we reported serological and generic studies in the non Jewish population of Mashhad as well as Neishabour. Seropositive rate of HTLV-1 in Mashhad is 3.0% (21of 694). DNA sequence were amplified by PCR directly from the fresh PRMCs of seropositive individuals from Mashhad. Phylogenetic analysis of the viral DNA sequence indicated that HTLV-1 in Mashhad belong to the cosmopolite clade. ATL and HAM/TSP is common in the northeast of Iran.

